# An Improved Human Evolution Optimization Algorithm for Unmanned Aerial Vehicle 3D Trajectory Planning

**DOI:** 10.3390/biomimetics10010023

**Published:** 2025-01-03

**Authors:** Xue Wang, Shiyuan Zhou, Zijia Wang, Xiaoyun Xia, Yaolong Duan

**Affiliations:** 1 School of Artificial Intelligence, Zhejiang Sci-Tech University, Hangzhou 310018, China; wangxuezstu@163.com; 2 School of Artificial Intelligence, Jiaxing University, Jiaxing 314001, China; 3 School of Information Engineering, Jiaxing Nanhu University, Jiaxing 314001, China; 4 School of Computer Science and Cyber Engineering, Guangzhou University, Guangzhou 510006, China; zijiawang@gzhu.edu.cn; 5 Technology Research and Development Centre, Xuelong Group Co., Ltd., Ningbo 315899, China; dyl7981@163.com

**Keywords:** improved human evolution optimization algorithm, logistic chaotic mapping, opposition-based learning strategy, guidance factor, adaptive *t*-distribution perturbation

## Abstract

To address the challenges of slow convergence speed, poor convergence precision, and getting stuck in local optima for unmanned aerial vehicle (UAV) three-dimensional path planning, this paper proposes a path planning method based on an Improved Human Evolution Optimization Algorithm (IHEOA). First, a mathematical model is used to construct a three-dimensional terrain environment, and a multi-constraint path cost model is established, framing path planning as a multidimensional function optimization problem. Second, recognizing the sensitivity of population diversity to Logistic Chaotic Mapping in a traditional Human Evolution Optimization Algorithm (HEOA), an opposition-based learning strategy is employed to uniformly initialize the population distribution, thereby enhancing the algorithm’s global optimization capability. Additionally, a guidance factor strategy is introduced into the leader role during the development stage, providing clear directionality for the search process, which increases the probability of selecting optimal paths and accelerates the convergence speed. Furthermore, in the loser update strategy, an adaptive *t*-distribution perturbation strategy is utilized for its small mutation amplitude, which enhances the local search capability and robustness of the algorithm. Evaluations using 12 standard test functions demonstrate that these improvement strategies effectively enhance convergence precision and algorithm stability, with the IHEOA, which integrates multiple strategies, performing particularly well. Experimental comparative research on three different terrain environments and five traditional algorithms shows that the IHEOA not only exhibits excellent performance in terms of convergence speed and precision but also generates superior paths while demonstrating exceptional global optimization capability and robustness in complex environments. These results validate the significant advantages of the proposed improved algorithm in effectively addressing UAV path planning challenges.

## 1. Introduction

Drone technology is rapidly advancing globally, serving as a vital component of strategic emerging industries. This progress fosters diversification and cross-sector integration, significantly contributing to low-altitude economic growth. As unmanned aerial vehicle (UAV) capabilities are enhanced and their application scenarios diversify, effective and safe flight path planning—aimed at avoiding obstacles, minimizing costs, and optimizing task efficiency—has emerged as a focal point of research in the UAV field.

UAV path planning seeks to identify a collision-free geometric path from the starting point to the destination [1]. However, this problem is widely regarded as NP-hard [2]. Currently, UAV path planning algorithms can be categorized into three primary types, model-based algorithms, sampling-based algorithms, and heuristic algorithms [3]. Model-based algorithms typically use precisely defined mathematical equations and constraints to address the path planning problem [4]. Masehian and Habibi utilized binary integer programming to plan the path of mobile robots in a three-dimensional environment [5]. Gong et al. proposed an adaptive pseudospectral method that transforms the optimal control problem into a nonlinear programming problem and applied an iterative convex programming algorithm to solve it [6]. Nevertheless, such algorithms exhibit limited representational capabilities in complex environments and incur high computational costs, which often restrict their practical applications. Sampling-based algorithms, such as the Dijkstra algorithm [7] and the A* algorithm [8], model the environment as a graph to find the optimal path and are commonly used for two-dimensional path planning. Nonetheless, they tend to be inefficient in handling complex environments. Rapidly exploring random trees (RRTs) [9] can quickly generate paths and are easy to implement and extend, but the paths generated are often tortuous, and the random nature of the waypoint generation does not ensure optimality. To enhance path quality, Karaman et al. proposed the RRT* algorithm [10], which improves path quality by reselecting parent nodes and reconstructing the random tree. Despite this, the new parent node search and rerouting processes also affect efficiency. The artificial potential field method [11] models the target area as an attractive force and obstacles as repulsive forces, simulating movement within a potential field to find the optimal path. However, this approach easily gets trapped in local optima when dealing with multi-target or multi-obstacle scenarios [12].

Given the NP-hard nature of path planning, metaheuristic algorithms have proven effective in addressing such challenges [13]. By introducing stochastic operators, metaheuristic methods treat path planning as an optimization problem and use heuristic functions to guide the search for the optimal solution. These algorithms have a relatively low complexity and can effectively tackle large-scale problems. Particularly, swarm intelligence algorithms, which mimic intelligent behaviors in nature to find optimal solutions, demonstrate excellent global search performances in UAV path planning within three-dimensional environments [14]. For instance, Deng et al. [15] proposed an enhanced Particle Swarm Optimization (PSO) algorithm specifically designed for 3D path planning. This enhanced algorithm combines PSO with genetic algorithms, incorporating dynamic inertia weights and integrating a sigmoid function to enhance genetic algorithm features such as crossover and mutation probabilities. The simulation results indicate that the improved PSO algorithm achieves remarkable path planning outcomes with a faster and more stable performance. Zhang et al. [16] augmented the Harris Hawks Optimization (HHO) algorithm using a Cauchy mutation strategy and adaptive weights, thereby increasing the population diversity and optimizing the UAV path planning results. Rajeev Kumar et al. [17] proposed the RLV-Grey Wolf Optimization algorithm, which utilizes reinforcement learning to adaptively control candidate operations for efficient path planning in 3D environments. Dewangan et al. [18] presented the Salp Swarm Algorithm (SSA), demonstrating its superior performance in 3D UAV route planning compared to other algorithms, enhancing the cost and time efficiency. Wang et al. [19] proposed an improved Tuna Swarm Optimization (TSO) algorithm featuring innovative strategies and showing an exceptional performance in flight path planning compared to other algorithms. Chen et al. [20] introduced an Opposition-Based Learning Artificial Bee Colony (OABC) algorithm, which incorporates individual abandonment probabilities and a target information entropy ratio model based on observation angles. Experimental results demonstrated that, compared to other methods, there was a significant reduction in the number of images obtained, and the efficiency of the 3D reconstruction was greatly improved. Wu et al. [21] proposed three optimization strategies to enhance the Moth Flame Optimization algorithm, including a chaos-based moth initialization, an adaptive weighted position update strategy, and an improved population diversity. The simulation results demonstrated the algorithm’s speed and optimality in UAV path planning problems.

Qadir et al. [22] adopted a new, dynamic, group-based collaborative optimization (DGBCO) algorithm to optimize the disaster assessment tasks of unmanned aerial vehicles. The key to this algorithm lies in using very few random variables, adjusting the parameters, and improving the search ability for global optimal solutions during the development and exploration stages through dynamic grouping methods. Subsequently, the Smart Flower Optimization (SFOA) algorithm was introduced to further optimize the path planning of unmanned aerial vehicles, with its performance evaluated in both a static environment and four dynamic environments. The results showed that the SFOA algorithm outperforms existing methods by achieving a reduction of 24.5% in transportation costs and 13.3% in computation time [23]. However, these metaheuristic algorithms often encounter challenges of slow convergence and a tendency to get trapped in local optima, which are particularly pronounced in high-dimensional spaces. Additionally, their performance is highly sensitive to parameter tuning, where slight adjustments can lead to the decreased reproducibility and reliability of the results, thereby increasing the workload and complexity to some extent. The Human Evolution Optimization Algorithm [24] is a novel, intelligent optimization algorithm proposed by Lian and Hui in 2024, characterized by high robustness, strong search capabilities, and a minimal need for parameter tuning. Inspired by human cultural and societal evolution, it seeks optimal solutions by simulating adaptive and innovative mechanisms in human society, exhibiting a strong potential and competitiveness in finding global optima. The introduction of the Human Evolution Optimization Algorithm provides new perspectives and solutions for UAV 3D path planning.

This paper introduces a novel and improved algorithm, termed the IHEOA, which is based on the Human Evolution Optimization Algorithm (HEOA) and incorporates multiple strategies to enhance its performance. To address challenges such as limited population diversity, premature convergence, and inadequate convergence precision, three strategies have been proposed. First, a strategy that combines opposition-based learning [25] with Logistic Chaotic Mapping [26] is employed to initialize the population, thereby improving the quality of the initial population. Second, a guidance factor is integrated into the leader position, updating the strategy during the development phase to assist individuals in navigating the solution space, thereby increasing the likelihood of selecting optimal paths and accelerating convergence speed. Lastly, an adaptive *t*-distribution perturbation [27] is incorporated into the position-updating strategy for loser individuals to enhance the algorithm’s local search capability.

The remainder of the paper is structured as follows: Section 1 provides a detailed description of three-dimensional environment modeling and the generation of flight paths using cubic uniform B-spline interpolation. Section 2 presents the multi-constraint cost model developed in this study. Section 3 briefly reviews the traditional HEOA and elaborates on the three proposed improvement strategies. Section 4 offers a comprehensive evaluation of the algorithm, based on twelve benchmark test functions to validate the effectiveness of the proposed strategies and the performance advantages of the IHEOA. Subsequently, simulation comparison experiments with various algorithms are conducted across three different environmental models to demonstrate the effectiveness and applicability of the IHEOA in 3D UAV path planning. Finally, Section 5 provides a conclusion.

## 2. 3D Path Planning Model for UAVS

### 2.1. Environment Modeling

The execution of UAV missions is crucial for determining operational efficiency, with path planning reliant on the specific environmental model utilized. A robust ecological model not only improves the accuracy of simulation experiments but also provides essential information for subsequent path optimization initiatives. To accurately simulate real-world UAV flight scenarios, this study selects typical natural mountain formations as the primary obstacle regions and employs a three-dimensional elevation map methodology for modeling. This approach effectively translates complex real terrain into mathematical models that are suitable for information processing. The mathematical model is described as follows:

(1) $$Z\left(x,y\right)=\sum_{i=1}^{N}h_{i}\exp\left[-{\left(\frac{x-x_{i}}{s_{xi}}\right)}^{2}-{\left(\frac{y-y_{i}}{s_{yi}}\right)}^{2}\right]$$

**Figure 1:**
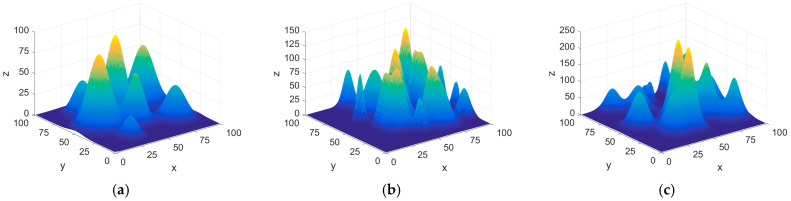
The three environmental models. (**a**) The 10-peak environment model. (**b**) The 20-peak environment model. (**c**) The 30-peak environment model.

In this model, (*x_i_*, *y_i_*) represents the center coordinates of the *i*-th peak, *h_i_* is the terrain parameter controlling the height of the peak, and *s_xi_* and *s_yi_* are the attenuation values along the *x*-axis and *y*-axis for the *i*-th peak, respectively, controlling the slope of the mountain. *N* denotes the total number of peaks in the mountainous environment. To comprehensively assess UAV performance across various natural terrains, this study utilizes three different environmental models featuring 10, 20, and 30 peaks, as illustrated in Figure 1.

These three terrain models represent different terrain undulations and obstacle distribution densities, aiming to simulate diverse natural environments. By gradually increasing the complexity of the terrain, we can systematically examine the obstacle avoidance ability and path planning effectiveness of drones in different difficulty environments, especially the impact of terrain undulations on drone mission execution. The 10-peak model represents relatively simple terrain and is mainly used to evaluate the performance of algorithms in relatively flat environments; the 20-peak and 30-peak models gradually increased the complexity of the terrain, simulating more complex and challenging natural environments. By comparing the performance of algorithms under different models, we can more accurately evaluate the robustness, scalability, and adaptability of the algorithms, and provide a strong basis for their further optimization and practical applications.

### 2.2. Generation of Flight Trajectory

**Figure 2:**
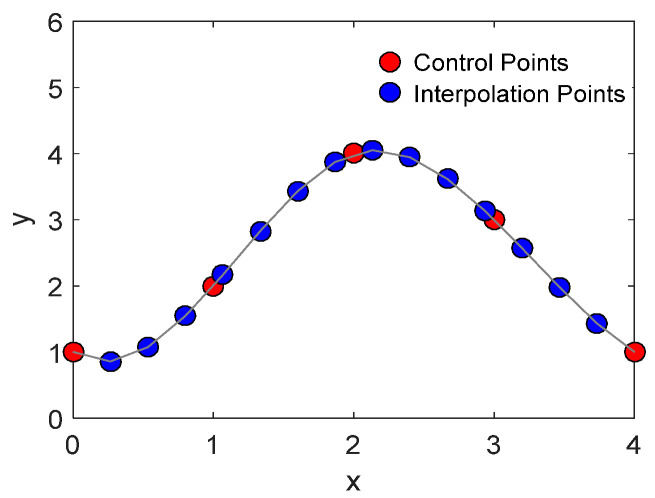
Cubic uniform B-Spline curve interpolation.

The generation of a UAV’s flight trajectory involves determining an ordered set of point coordinates, referred to as control points, which serve as the foundation for the UAV’s spatial movements. These control points define the shape of the flight path and are essential for path optimization. By applying cubic uniform B-spline curve interpolation to these control points, it is possible to flexibly adjust the curve shape between them, thus generating a smooth and continuous flight trajectory, as illustrated in Figure 2.

For a set of control points *P*_0_, *P*_1_, …, *Pn*, the general equation for cubic uniform B-spline curve interpolation can be expressed as follows:

(2) $$C\left(t\right)=\sum_{i=0}^{n}P_{i}\cdot N_{i,3}\left(t\right)$$

In this equation, *P_i_* represents the coordinates of the *i*-th control point, while *N_i,_*_3_(*t*) signifies the cubic B-spline basis function corresponding to that control point. The cubic uniform B-spline basis functions are defined recursively as follows:

(a) Zeroth-order basis function (0th order).
  (3) $$N_{i,0}\left(t\right)=\left\{\begin{array}{l} 1,t_{i}\leq t\leq t_{i+1}\\ 0,otherwise \end{array}\right.$$
(b) First-order basis function (1st order).
  (4) $$N_{i,1}\left(t\right)=\frac{t-t_{i}}{t_{i+1}-t_{i}}N_{i,0}\left(t\right)+\frac{t_{i+2}-t}{t_{i+2}-t_{i+1}}N_{i+1,0}\left(t\right)$$
(c) Second-order basis function (2nd order).
  (5) $$N_{i,2}\left(t\right)=\frac{t-t_{i}}{t_{i+1}-t_{i}}N_{i,1}\left(t\right)+\frac{t_{i+3}-t}{t_{i+3}-t_{i+1}}N_{i+1,1}\left(t\right)$$
(d) Third-order basis function (3rd order).
  (6) $$N_{i,3}\left(t\right)=\frac{t-t_{i}}{t_{i+1}-t_{i}}N_{i,2}\left(t\right)+\frac{t_{i+4}-t}{t_{i+4}-t_{i+1}}N_{i+1,2}\left(t\right)$$

Each stage of the basis function, starting from the zeroth order, contributes to building up the cubic B-spline used for the interpolation of the control points. This process ensures the desired smoothness and continuity of the flight trajectory.

## 3. Multi-Constraint Path Cost Model

In UAV path planning, a multi-constraint path cost model is essential for ensuring flight safety and efficiency. This model incorporates various constraints and adjusts the corresponding control variables, *c*_1_, *c*_2_, and *c*_3_, to compute the fitness function of a given path. This process optimizes the UAV’s flight trajectory, facilitating the efficient and safe completion of missions. This section presents a comprehensive analysis of several factors, including altitude constraints, positional constraints, maximum turning angle constraints, maximum climb angle constraints, and path length costs.

### 3.1. Altitude Constraint

The altitude constraint ensures that the UAV maintains a specific safe altitude range during flight to avoid terrain obstacles, buildings, and other potential risks. This constraint is particularly crucial in environments with a complex terrain. This paper formalizes the altitude constraint model as follows:

(7) $$Z_{i}>Z\left(x_{i},y_{i}\right),i=1,2,\ldots ,N$$

where *Z_i_* denotes the UAV’s flying altitude, and Z(*x_i_*, *y_i_*) represents the terrain height at the coordinate point (*x_i_*, *y_i_*). When the UAV’s flight altitude is less than or equal to the terrain height, the control variable, *c*_1_, is activated, resulting in an increase in the fitness penalty. This weighted penalty significantly enhances the algorithm’s responsiveness and adaptability in complex environments.

### 3.2. Position Constraint

Position constraints are a critical mechanism used to restrict a UAV’s operations to a designated area during mission execution. The aim is to ensure that the UAV’s flight path aligns with real-world geographical conditions and meets the specific requirements of the mission, thereby effectively avoiding potential collision risks in unknown environments. This paper formalizes the position constraint model as follows:

(8) $$\left\{\begin{array}{l} 0<x_{i}<x_{\max}\\ 0<y_{i}<y_{\max}\\ 0<z_{i}<z_{\max} \end{array}\right.,i=1,2,\ldots ,N$$

where *x*_max_, *y*_max_, and *z*_max_ are the maximum values in each dimension of the solution space. When a path point (*x_i_*, *y_i_*, or *z_i_*) exceeds these boundaries, the corresponding control variable, *c*_2_, is activated, applying an additional fitness penalty to encourage path correction.

### 3.3. Maximum Turning Angle Constraint

In UAV path planning, limiting the maximum turning angle is a key factor for ensuring both flight safety and navigational efficiency. Neglecting this constraint can result in significant discrepancies between paths obtained in the dynamic and the static planning stages and may impact the UAV’s maneuverability and stability. By thoroughly considering factors such as a sufficient number of orderly points in the flight trajectory, reduced distances between nodes, and shorter flight times—alongside the assumption of uniform motion—it becomes feasible to calculate the turning angle with greater accuracy. This approach enhances the UAV’s flexibility and stability in complex environments.

Specifically, assuming that the velocity projection at node *i* on the horizontal plane is *V_i_*, and at node *i +* 1 it is *V_i+_*_1_, the formula for calculating the turning angle *θ* is

(9) $$\theta =\arccos\left(\frac{V_{i}^{T}\cdot V_{i+1}}{\left\|V_{i}\cdot V_{i+1}\right\|}\right)\leq \theta_{\max}$$

### 3.4. Maximum Climb Angle Constraint

In addition to the maximum turning angle, UAVs are also subject to constraints imposed by the maximum climb angle, which regulates the rate of ascent and descent. This constraint is crucial for ensuring smooth and safe flight operations. The formula for calculating the climb angle *γ* is as follows:

(10) $$\gamma =\arctan\left(\frac{\left|z_{i}-z_{i+1}\right|}{\sqrt{{\left(x_{i}-x_{i+1}\right)}^{2}+{\left(y_{i}-y_{i+1}\right)}^{2}}}\right)\leq \gamma_{\max}$$

In this study, when the constraints for the maximum turning angle and the maximum climb angle are not met, the system activates control variable *c*_3_. This mechanism significantly enhances the algorithm’s responsiveness and adaptability in complex environments by increasing the weight of the fitness penalty. Such dynamic adjustments ensure that the UAV can efficiently and safely execute a range of tasks in varying conditions, further optimizing the decision-making process during path planning.

### 3.5. Path Length Cost

The path length cost is primarily used to quantify the actual flying distance of a UAV from the starting point to the endpoint, with the objective of minimizing the flight path length to achieve the dual optimization of time and energy. The path length, *L*, can be calculated using the following formula:

(11) $$L=\sum_{i=1}^{N}d_{i}=\sum_{i=1}^{N}\sqrt{{\left(x_{i}-x_{i+1}\right)}^{2}+{\left(y_{i}-y_{i+1}\right)}^{2}+{\left(z_{i}-z_{i+1}\right)}^{2}}$$

where *d_i_* represents the Euclidean distance between two adjacent points in the path, and *N* denotes the total number of sampled points on the path. Minimizing this cost function not only reduces the time required for flight but also lowers energy consumption, thereby enhancing the economic efficiency of mission execution.

### 3.6. Objective Function

In the process of flight path planning, the path cost is closely related to the degree to which the constraints are satisfied. In this study, when the planned flight path fully satisfies all the established constraints, the cost is defined by the length of the flight path, thereby reflecting optimal flight efficiency. Conversely, when there are unsatisfied constraints, the weights of the relevant control variables are appropriately increased to enhance the penalty for these unsatisfied constraints. This mechanism encourages subsequent iterations of the flight path to more closely adhere to all constraints. The expressions for each constraint control variable are as follows:

(12) $$c_{1}=\left\{\begin{array}{cc} 1000, & Z_{i}<Z\left(x_{i},y_{i}\right)\\ 1, & Z_{i}\geq Z\left(x_{i},y_{i}\right) \end{array}\right.$$

(13) $$c_{2}=\left\{\begin{array}{cc} 1.5, & \left(x_{i},y_{i},z_{i}\right)\notin P\\ 1, & \left(x_{i},y_{i},z_{i}\right)\in P \end{array}\right.$$

(14) $$c_{3}=\left\{\begin{array}{cc} 1, & \left|\gamma \right|\leq \gamma_{\max}\;and\;\left|\theta \right|\leq \theta_{\max}\\ 2, & \left|\gamma \right|>\gamma_{\max}\;and\;\left|\theta \right|>\theta_{\max} \end{array}\right.$$

where *c*_1_, *c*_2_, and *c*_3_ are the control variables for altitude constraint, position constraint, and the constraints of the maximum turning angle and maximum climb angle, respectively; *P* represents the solution space of the optimization problem. The objective function expressions based on each constraint are defined as follows:

(15) $$M\mathrm{in}\;F=c_{1}\times c_{2}\times c_{3}\times L$$

In this formulation, *L* represents the path length. This design facilitates the weighting of various control variables, thereby increasing the cost when the constraints are not satisfied. Consequently, this mechanism guides the path planning process toward a more comprehensive fulfillment of all constraints.

Here, the design of the control variable values takes into account the extent to which different constraints affect the path. Specifically, *c*_1_ is set to 1000 to emphasize the critical importance of altitude for flight safety; *c*_2_ is set to 1.5, reflecting its significant, though less critical, impact on the path; and *c*_3_ is set to 2, accounting for the physical constraints imposed by turning and climb angles on the flight path. This balanced strategy facilitates adaptive adjustments in cost evaluation, ensuring that different constraints are effectively incorporated into the path planning process and optimizing the UAV flight path to strike a balance between safety and efficiency. Consequently, the proposed path cost model significantly enhances UAV path planning optimization, ensuring that flight missions are both safe and efficient.

## 4. Improved Human Evolutionary Optimization Algorithm for UAV Path Planning

### 4.1. Human Evolutionary Optimization Algorithm

The Human Evolutionary Optimization Algorithm (HEOA) is a metaheuristic algorithm that is grounded in the principles of biological evolution and natural selection. It addresses complex optimization problems by simulating human evolutionary and adaptive processes, dividing the global search into exploration and exploitation phases.

#### 4.1.1. Population Initialization

In the population initialization phase, the HEOA uses Logistic Chaotic Mapping to generate diverse individuals. Initially, a Logistic Chaotic Mapping generates the initial population, which is then mapped according to the boundaries of the search space. The specific mapping expressions are as follows:

(16) $$x_{i}=\alpha \cdot x_{i-1}\cdot \left(1-x_{i-1}\right),0\leq x_{0}\leq 1,i=1,2,\ldots ,N,\alpha =4$$

(17) $$x_{i}^{0}=lb+\left(ub-lb\right)\cdot x_{i}$$

where *x_i_* represents the position of the *i*-th individual, *N* denotes the population size, and *α* is the coefficient of the Logistic Chaotic Mapping. $x_{i}^{0}$ is the specific position of *x_i_* mapped within the search space, where *lb* and *ub* represent the lower and upper bounds of the search space, respectively.

#### 4.1.2. Exploration Phase

In the exploration phase (the first quarter of the iterations), the HEOA incorporates dynamic exploration elements to improve the randomness and dispersion of the search process. A jump strategy, inspired by image compression, is employed to effectively prevent excessive searching near local optima. Simultaneously, the Levy search strategy is utilized to enhance the global search capability, improving the algorithm’s ability to locate the global optimum. Additionally, an adaptive weight sampling method controls the search step size to improve search precision and efficiency. Combined, these strategies enable the effective exploration of unknown spaces. The specific position update formula is as follows:

(18) $$\begin{array}{l} X_{i}^{t+1}=\beta \cdot \left(1-\frac{t}{Max_{iter}}\right)\cdot \left(X_{i}^{t}-X_{best}\right)\cdot Levy\left(\dim\right)\\ +X_{best}\cdot \left(1-\frac{t}{Max_{iter}}\right)+\left(X_{mean}^{t}-X_{best}\right)\cdot floor\left(\frac{rand}{f_{jump}}\right)\cdot f_{jump} \end{array}$$

where *β* is an adaptive function, *t* denotes the iteration count, *dim* represents the dimension of the problem, $X_{i}^{t}$ denotes the current position, and $X_{i}^{t+1}$ indicates the subsequent updated position. *X_best_* corresponds to the best position explored thus far, while $X_{mean}^{t}$ represents the average position of the current population. The operation *floor* refers to rounding down. *Levy* indicates the Levy distribution, *f_jump_* is the jump factor, and *rand* is a random number in the range [0, 1].

Here, the average ranking, $X_{mean}^{t}$, represents the current population’s average position, and its expression is

(19) $$X_{mean}^{t}=\frac{1}{N}\sum_{k=1}^{N}X_{t}^{k}$$

The adaptive function, *β,* is responsible for adjusting the parameters based on the iteration count and the current position, and its expression is

(20) $$\beta =0.2\cdot \left(1-\frac{t}{Max_{iter}}\right)\cdot \left(X_{i}^{t}-X_{mean}^{t}\right)$$

The Levy distribution models the complexity of human knowledge acquisition and the spiral development characteristic during the exploration phase, and its expression is

(21) $$\left\{\begin{array}{l} Levy\left(D\right)=\frac{\mu \cdot \sigma }{{\left|v\right|}^{\frac{1}{\gamma }}}\\ \mu \sim N\left(0,N\right)\\ \nu \sim N\left(0,N\right)\\ \sigma =\left(\frac{\Gamma \left(1+\gamma \right)\cdot \sin{\left(\frac{\pi \gamma }{2}\right)}^{\gamma +1}}{\Gamma \left(\frac{1+\gamma }{2}\right)\cdot \gamma \cdot 2^{\frac{1+\gamma }{2}}}\right) \end{array}\right.$$

The jump factor, *f_jump_*, quantifies the extent of the jump, aiming to enhance the dispersion of search positions, and its expression is

(22) $$f_{jump}=\frac{\left(lb\left(1\right)-ub\left(1\right)\right)}{\delta },\delta \in \left[1000,2000\right]$$

#### 4.1.3. Development Phase

In the development phase, the HEOA classifies individuals into four distinct roles: leaders, explorers, followers, and laggards. Each role employs a specific search strategy to collaboratively explore the global optimal solution.

The first 40% of individuals in the pre-adaptation phase are designated as leaders, who are primarily responsible for exploring the superior domains of human development using existing knowledge. Leaders select an appropriate update strategy based on the complexity of the situation. The position update expression for leaders is given by

(23) $$X_{i}^{t+1}=\left\{\begin{array}{cc} \omega \cdot X_{i}^{t}\cdot \exp\left(\frac{-t}{rand\cdot Max_{iter}}\right), & R<A\\ \omega \cdot X_{i}^{t}+Rn\cdot ones\left(1,\dim\right), & R\geq A \end{array}\right.$$

In this equation, *Rn* denotes a random number that follows a normal distribution, while the function *ones* (1, *dim*) generate a row vector containing *dim* elements, each equal to one. The random number *R* is constrained within the range [0, 1] and reflects the complexity of the context related to the leader. The variable *A* represents the assessment value of the situation, which is set to 0.6 in this study. Based on the complexity of the specific position’s situation, leaders select an appropriate search strategy. The knowledge acquisition difficulty coefficient is denoted as *ω*, which gradually decreases as development progresses. The expression for this coefficient is as follows:

(24) $$\omega =0.2\cdot \cos\left(\frac{\pi }{2}\left(1-\frac{t}{Max_{iter}}\right)\right)$$

Explorers are individuals ranked between the top 40% and 80% in terms of fitness within the population. They play a crucial role in exploring uncharted territories to discover the global best solution. The strategy they employ is expressed as follows:

(25) $$X_{i}^{t+1}=Rn\cdot \exp\left(\frac{X_{worst}^{t2}-X_{i}^{t2}}{i^{2}}\right)$$

where $X_{worst}^{t}$ denotes the position of the least fit individual in the population during the *t^th^* iteration.

Followers are individuals ranked between the top 80% and 90% of the population, based on fitness. They search by following in the footsteps of the leaders, and their strategy is expressed as follows:

(26) $$X_{i}^{t+1}=X_{i}^{t}+\omega \cdot Rd\cdot \left(X_{best}^{t}-X_{i}^{t}\right)$$

where $X_{best}^{t}$ represents the position of the fittest individual in the population during the iteration, and *Rd* is a random number within the range [1, *dim*].

Conversely, laggards refer to those individuals performing poorly in the current environment. To optimize the population, laggards are eliminated and replaced through reproduction in areas conducive to human development. The population replacement formula is given by

(27) $$X_{i}^{t+1}=X_{best}+\left(X_{best}-X_{i}^{t}\right)\cdot Rn$$

### 4.2. Improved Human Evolution Optimization Algorithm

#### 4.2.1. Integration of Logistic Chaotic Mapping and Opposition-Based Learning Strategy in Population Initialization

In the HEOA optimization algorithm framework, the initial population is generated using Logistic Chaotic Mapping. Although the Logistic Chaotic Mapping displays various behaviors (including stability, periodicity, and chaos) in a one-dimensional space, particularly when the parameter *α* is set to four in equation (16), its output shows comprehensive chaotic properties within the range of [0, 1] [28,29]. Compared with traditional random number generators, Logistic Chaotic Mapping has the advantage of generating complex and uniformly distributed random number sequences while maintaining determinism and repeatability. However, in this study, when the point coordinates are generated using the Logistic Chaotic Mapping and mapped to the three-dimensional space, it is observed that the distribution of the point coordinates in the space is not uniform. This unevenness may have a direct impact on the generation of the initial solution (point coordinate sequence), thereby limiting the effectiveness and comprehensiveness of the algorithm in the process of seeking the optimal solution. At the same time, this also limits the ability of the algorithm to fully explore the search space, to a certain extent, and increases the risk of early convergence to a local optimal solution.

In order to make the generated solutions more widely and uniformly distributed in the search space and obtain higher quality initial solutions, this study combines Logical Chaotic Mapping with an opposition-based learning strategy to propose a novel initialization strategy. This strategy generates the initial population of individuals using the Logistic Chaotic Mapping and simultaneously creates their opposite solutions. Subsequently, a fitness evaluation is then conducted for twice the number of individuals, allowing for the selection of high-quality individuals that satisfy the population size requirements. This approach significantly improves the quality of the initial population’s distribution within the search space, enhances the algorithm’s global search capability, and accelerates the convergence speed during the exploration phase to some extent. The mathematical expression for the opposition-based learning strategy is as follows:

(28) $$\left\{\begin{array}{l} x_{i}=\alpha \cdot x_{i-1}\cdot \left(1-x_{i-1}\right),0\leq x_{0}\leq 1,i=1,2,\ldots ,N,\alpha =4\\ x_{i}^{0}=lb+\left(ub-lb\right)\cdot x_{i}\\ x_{i}^{1}=\left(lb-ub\right)-x_{i} \end{array}\right.$$

where $x_{i}^{1}$ is the opposite solution corresponding to each initial solution $x_{i}^{0}$, and *lb* and *ub* are the lower and upper bounds of the search space, respectively.

**Figure 3:**
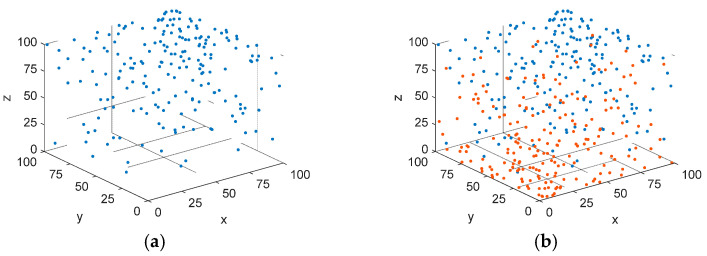
Comparative distribution of the initial population under two strategies. (**a**) Logistic Chaotic Mapping. (**b**) Logistic Chaotic Mapping with integrated opposition-based strategy.

A comparative analysis of the initial population, generated by the Logistic Chaotic Mapping and the Logistic Chaotic Mapping combined with the opposition-based learning strategy, demonstrates that the improved initialization strategy exhibits superior stability and uniformity in distribution, as depicted in Figure 3. In the figure below, the blue points represent control points generated by the Logistic Chaotic Mapping, while the red points indicate control points generated by the opposition-based learning strategy.

#### 4.2.2. Guidance Factor

During the development phase of the HEOA, leaders determine the particle position update strategy by comparing a random value, *R,* with a threshold, *A.* When the random value, *R*, is less than the threshold, *A*, particles adopt an update strategy that combines randomness with global exploration. This approach facilitates exploration within a broader search space and effectively prevents entrapment in local optima. Conversely, when *R* is greater than or equal to *A*, although the second strategy retains some global exploration capability by introducing random noise, particles may experience slower convergence in high-dimensional spaces. This is notably the case when the initial positions are far from the target, requiring more iterations to approach the target point.

To overcome this challenge, this study introduces a guidance factor to accelerate particle movement toward the target position, thus speeding up the convergence process. By independently calculating a guidance factor for each particle across different spatial dimensions, particles can make more precise and flexible adjustments during updates. The mathematical expression for the guidance factor in each dimension is as follows:

(29) $$G\_factor_{i}=\left\{\begin{array}{l} G\_factor_{i}^{x}=1-\frac{\left|p_{i}^{x}-g\_pos_{x}\right|}{d}\\ G\_factor_{i}^{y}=1-\frac{\left|p_{i}^{y}-g\_pos_{y}\right|}{d}\\ G\_factor_{i}^{z}=1-\frac{\left|p_{i}^{z}-g\_pos_{z}\right|}{d} \end{array}\right.$$

where $G\_factor_{i}^{x}$, $G\_factor_{i}^{y}$, and $G\_factor_{i}^{z}$ denote the guidance factors along the x, y, and z axes for the *i^th^* particle, ($p_{i}^{x},p_{i}^{y},p_{i}^{z}$) represents the spatial position of the *i^th^* particle, (*g_pos_x_*, *g_pos_y_*, *g_pos_z_*) indicates the target position for path planning, and *d* is the Euclidean distance from the initial position to the target position.

Moreover, this study develops a directional search strategy that combines a guidance factor with random perturbations. This strategy enables particles to effectively update their positions in space with variable step sizes that are determined by the distance from their current position to the target position during the search for the optimal solution. This flexible step size adjustment mechanism not only prevents particles from falling into local optima but also significantly enhances the exploration capability of the search space. The position update strategy for leaders, with the incorporation of the guidance factor, is detailed below.

(30) $$X_{i}^{t+1}=\left\{\begin{array}{cc} \omega \cdot X_{i}^{t}\cdot \exp\left(\frac{-t}{rand\cdot Max_{iter}}\right), & R<A\\ \omega \cdot X_{i}^{t}+G\_factor_{i}\cdot Rn, & R\geq A \end{array}\right.$$

#### 4.2.3. Adaptive *t*-Distribution Perturbation Strategy

**Figure 4:**
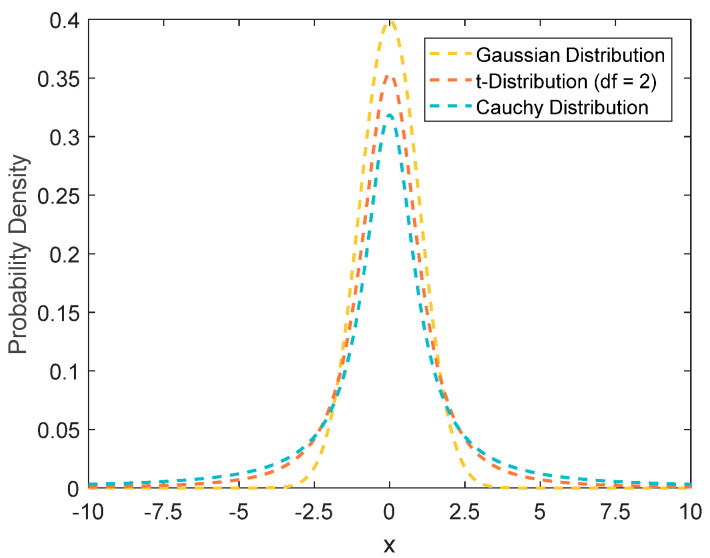
Illustration of Gaussian distribution, *t*-distribution, and Cauchy distribution.

The *t*-distribution, commonly referred to as Student’s *t*-distribution, is a probability distribution dependent upon the degrees of freedom parameter *n*. The value of *n* directly influences the shape of the *t*-distribution curve: a smaller *n* results in a flatter curve, whereas a larger *n* produces a steeper curve. As *n*→∞, the *t*-distribution approaches the standard Gaussian distribution *N*(0,1); conversely, when *n* = 1, it degenerates into the standard Cauchy distribution *C*(0,1). Therefore, the standard Gaussian and Cauchy distributions are two boundary special cases of the *t*-distribution. The probability density functions of these distributions are illustrated in Figure 4.

In optimization algorithms, the Cauchy mutation operator enhances the global exploration capability while maintaining population diversity. Conversely, the Gaussian mutation operator strengthens the local search ability, ensuring the convergence speed in the later stages of evolution. The *t*-distribution synthesizes the advantages of both the Cauchy and Gaussian distributions, allowing for a balance between the global and local search capabilities by adjusting the degrees of freedom parameter, *n* (ranging from one to infinity). Consequently, this study applies an adaptive *t*-distribution mutation operator within the position update strategy for non-leaders, setting its degrees of freedom to the current iteration number, *iter*. This approach endows the algorithm with stronger global exploration capabilities in the early iterations and excellent local exploitation abilities in the later iterations, thus enhancing the convergence speed of the algorithm significantly. The specific position update method is as follows:

(31) $$X_{i}^{t+1}=X_{best}+\left(X_{best}-X_{i}^{t}\right)\cdot t\left(iter\right)$$

where *t*(*iter*) represents the adaptive *t*-distribution perturbation, which dynamically adjusts with the iteration number.

#### 4.2.4. Overall Framework for Path Planning, Based on the IHEOA

Compared to the HEOA, the proposed IHEOA combines the above three innovative strategies to improve the convergence accuracy and global search ability of the algorithm, solving the problems of insufficient convergence accuracy and susceptibility to locally optimal solutions in the original HEOA for UAV 3D path planning. Firstly, in the initialization stage of the population, an opposition-based learning strategy is adopted, combined with Logistic Chaotic Mapping for initialization, in order to obtain spatially uniformly distributed and high-quality initial solutions, avoiding falling into local optima. Secondly, in the stage of leader position update, the guidance factor is added to accelerate the convergence process and improve the accuracy and efficiency of leader position update. Finally, in the loser update strategy, an adaptive *t*-distribution perturbation strategy is introduced to enhance the local search capability and avoid algorithm stagnation in the local optimal region. Through these improvements, the IHEOA exhibits a stronger performance in drone path planning, effectively improving convergence accuracy and enhancing global search capabilities. The steps for the path planning process using this algorithm are as follows:

Step 1: Model the environment, initialize the algorithm parameters, and determine the starting and target positions of the unmanned aerial vehicle (UAV).

Step 2: Generate an initial population using Logistic Chaotic Mapping with integrated opposition-based learning. Paths are generated through cubic B-spline interpolation, and the fitness of the individuals in the population is calculated.

Step 3:During the exploration phase (representing one-fourth of the total iterations), employ specific strategies to explore the solution space to obtain better individual and fitness values while recording the flight paths.

Step 4: In the development phase, apply relevant position update strategies for individuals based on their roles. Additionally, integrate a position update strategy based on a combined guidance factor for leader individuals, and incorporate an adaptive *t*-distribution-based position update strategy for the optimization of lagging individuals.

Step 5: Verify whether the maximum number of iterations has been reached. If it has not, return to Step 2; otherwise, conclude with the identified optimal solution.

**Figure 5:**
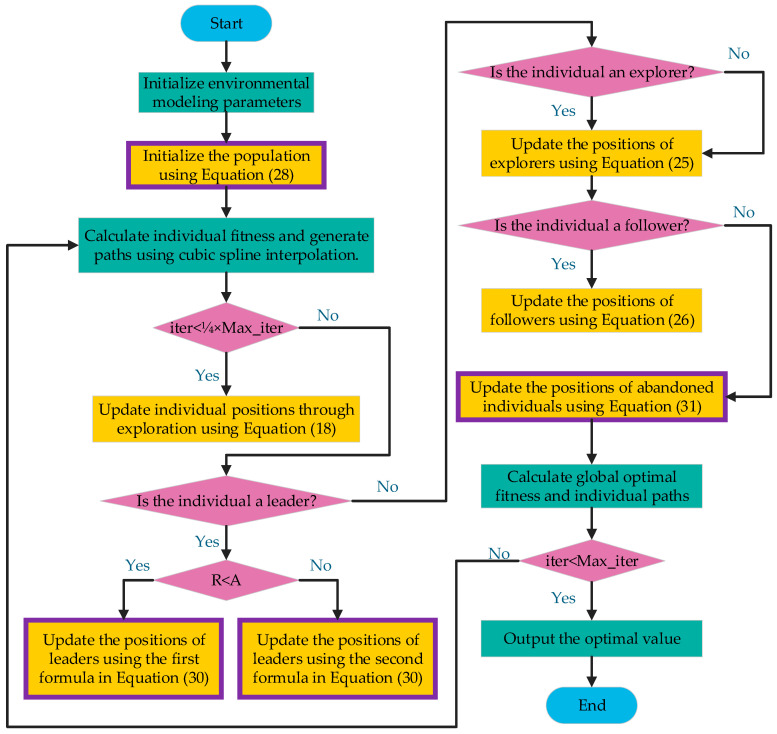
Flowchart of the IHEOA.

The algorithm flowchart is shown in Figure 5, and the part introducing strategies into the IHEOA has been highlighted in purple in the flowchart.

## 5. Simulation and Validation

### 5.1. Algorithm Testing

Algorithm test functions are important tools for evaluating algorithm performance, and performance evaluation indicators can more reliably measure the advantages and disadvantages of algorithms. In order to verify the effectiveness of the three strategies proposed by the Human Evolution Optimization Algorithm (HEOA), we selected twelve benchmark functions from the CEC test function library for the comparative analysis [30,31]. These benchmark functions include six unimodal functions and six multimodal functions, covering different types of problem characteristics. Detailed information regarding the test functions is presented in Table 1.

**Figure 6:**
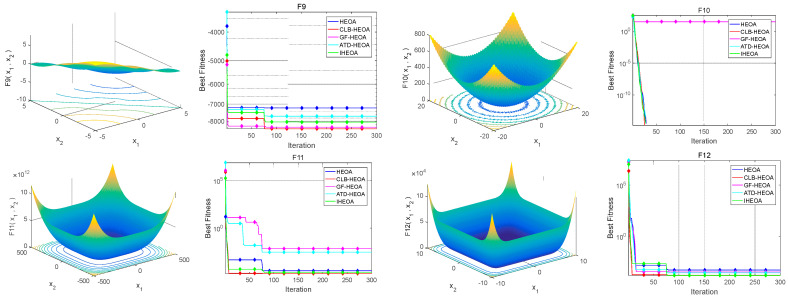

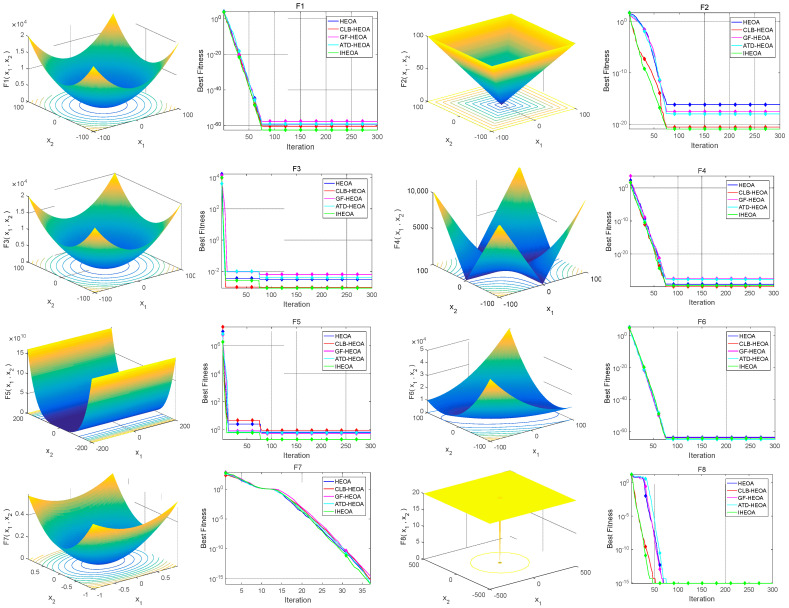
Convergence behavior of different algorithms.

In this experiment, the population size for all algorithms was set at 50, and the maximum number of iterations was limited to 300. To ensure the reliability and stability of the results, each algorithm was executed independently 30 times. The performance was evaluated using several metrics, including the maximum value (Max), minimum value (Min), mean value (Mean), and standard deviation (Std) to assess the optimization performance. The results of the different algorithms in the test functions are presented in Table 2, while the corresponding convergence behavior is illustrated in Figure 6. The algorithms tested in this study include

1. The traditional Human Evolutionary Optimization Algorithm (HEOA);
2. The HEOA with each of the three strategies applied individually:
  (a) Chaotic Logistic and Backward Learning Human Evolutionary Optimization Algorithm (CLB-HEOA);
  (b) Guidance Factor Human Evolutionary Optimization Algorithm (GF-HEOA);
  (c) Adaptive *t*-distribution Human Evolutionary Optimization Algorithm (ATD-HEOA).
3. The Improved Human Evolutionary Optimization Algorithm (IHEOA), which integrates all three strategies.

The experimental environment consisted of an Intel (R) Core (TM) i5-7300HQ CPU @ 2.50GHz with 8.00 GB of memory on a Windows 10 operating system, and the algorithms were implemented using MATLAB 2016a.

In the evaluation of optimization algorithms, unimodal functions (F_1_–F_6_) and multimodal functions (F_7_–F_12_) are used to assess the exploitation and exploration capabilities of different algorithms. Unimodal functions primarily evaluate an algorithm’s ability to quickly converge to the global optimum in deterministic environments, while multimodal functions test the algorithm’s ability to escape local optima and identify the global optimum. In this experiment, smaller test results signify a superior algorithm performance. For each test function, we use the execution results of the HEOA as a benchmark and compare the performance of the other four algorithms. The comparison indicators include maximum value (Max), minimum value (Min), mean value (Mean), and standard deviation (Std). In order to facilitate a more intuitive evaluation of the advantages and disadvantages of the algorithm, we bold the best function test results (i.e., smaller values) in each indicator to highlight the performance of the algorithm on different performance indicators.

The experimental results indicate that the CLB-HEOA performs exceptionally well in unimodal functions F_1_-F_4_ and F_6_, as well as on multimodal functions, particularly excelling in achieving lower minimum (Min) and mean (Mean) values compared to the traditional HEOA. This suggests that the CLB-HEOA has a significant advantage in converging to smaller errors. Its superior performance in terms of maximum (Max) and standard deviation (Std) further validates that the integration of backward learning strategies with Logistic Chaotic Mapping in the CLB-HEOA enhances diversity and depth in exploring the solution space, effectively avoiding local optima.

The GF-HEOA demonstrates lower standard deviations (Std) in unimodal functions F_1_-F_3_ and F_6_ and multimodal functions F_7_ and F_10_, indicating greater stability in the results. From functions F_2_, F_3_, F_7_, and F_9_, it is evident that the GF-HEOA also shows better performance in the maximum (Max) and mean (Mean) values, achieving higher precision in convergence. Conversely, the ATD-HEOA displays stable convergence characteristics in unimodal functions F_2_, F_3_, F_5_, and F_6_ and multimodal functions, achieving a higher convergence precision and showcasing superior optimization capabilities.

Notably, the IHEOA excels across nearly all test functions, with its maximum, minimum, mean, and standard deviation outperforming the other algorithms, highlighting the significant advantages of strategy integration. For instance, in function F_1_, the IHEOA attains a minimum value of 3.3071 × 10^−63^ and a mean of 1.1365 × 10^−50^, which illustrates its exceptional optimization performance and stability. By integrating multiple strategies, the IHEOA effectively capitalizes on the strengths of each strategy, resulting in significant performance enhancements and demonstrating stronger global search capabilities and higher convergence precision.

In summary, the performance of the various algorithms across different test functions clearly illustrates the substantial impact of strategies on algorithm performance. Through the integration of multiple strategies, the IHEOA demonstrates superior performance in both unimodal and multimodal functions, proving the effectiveness and advantages of this integration. This also indicates that the selection and integration of strategies are crucial factors in enhancing algorithm performance, further highlighting the innovation and practicality of this research in optimization algorithm design.

### 5.2. Simulation Calculation and Validation

To validate the effectiveness of the improved HEOA in UAV path planning problems, this study generates environmental models with 10, 20, and 30 peaks in a three-dimensional space with dimensional ranges of [0, 100]. An increased number of peaks indicates greater complexity in path planning. The experimental setup designates starting coordinates as [1, 1, 1] and target coordinates as [100, 100, 80], with population sizes of 50, 100, and 100 and iteration counts of 60, 100, and 100, respectively. In the experiments, statistics were collected on the best outcomes (Best), mean values (Mean), variance (Std), number of invalid paths (Invnum-path), valid path rates (Valid-rate), and the optimal fitness achieved by the HEOA and the IHEOA during the first quarter of iterations. To mitigate stochastic error, the IHEOA was compared with the HEOA, the Genetic Algorithm (GA), Particle Swarm Optimization (PSO), Grey Wolf Optimizer (GWO), and the Artificial Bee Colony algorithm (ABC) through trajectory simulation. Each algorithm was independently executed 30 times in distinct three-dimensional environmental models. The detailed experimental results are presented in Table 3.

**Figure 7:**
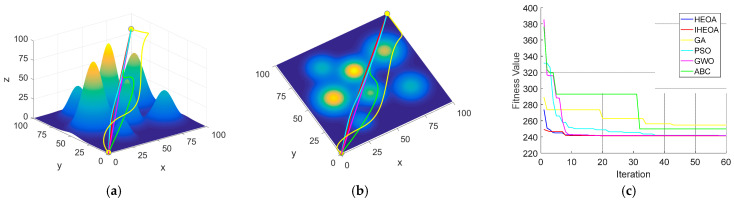
Comparative simulation diagrams for the 10-peak model. (**a**) Front view of the 10-peak model. (**b**) Top view of the 10-peak model. (**c**) Algorithm convergence comparison chart.

The 10-peak environmental model, which presents relatively low planning difficulty, serves as an appropriate benchmark for assessing the global optimization capabilities of the algorithm. Under the condition of 60 iterations, all the algorithms successfully generated valid paths. However, the IHEOA attained the lowest optimal fitness, with its average fitness reduced by 10.5%, 0.5%, 1.2%, 14.7%, and 0.4% compared to the GA, PSO, GWO, ABC, and HEOA, respectively. As shown in Figure 7, the path generated by the IHEOA is smoother with fewer inflection points and demonstrates faster convergence.

**Figure 8:**
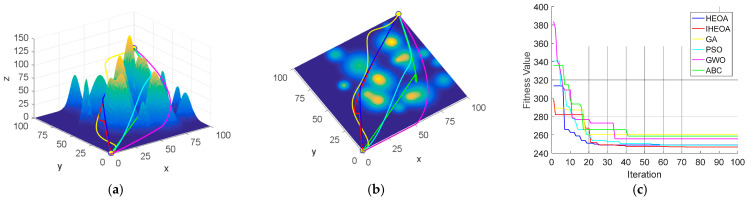
Comparative simulation diagrams for the 20-peak model. (**a**) Front view of the 20-peak model. (**b**) Top view of the 20-peak model. (**c**) Algorithm convergence comparison chart.

In the 20-peak environmental model, which presents moderate planning difficulty with 100 iterations, the IHEOA demonstrated a consistent advantage by achieving the lowest optimal fitness. Its average fitness was reduced by 13.5%, 1.2%, 11.3%, 30.5%, and 2% compared to the aforementioned algorithms. Figure 8 illustrates that the IHEOA exhibits superior optimization performance and convergence characteristics.

**Figure 9:**
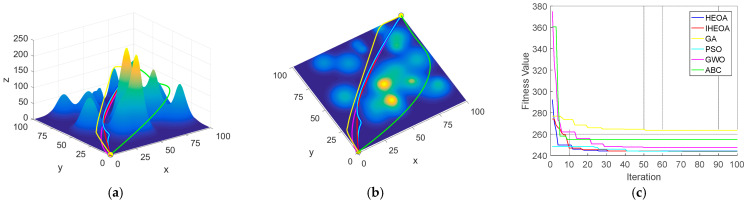
Comparative simulation diagrams for the 30-peak model. (**a**) Front view of the 30-peak model. (**b**) Top view of the 30-peak model. (**c**) Algorithm convergence comparison chart.

The robustness of various algorithms was assessed in the highly complex 30-peak environmental model. The IHEOA demonstrated superior precision in terms of optimal fitness, with average fitness reduced by 16.1%, 2.2%, 11.4%, 40.2%, and 1.1% compared to the GA, PSO, GWO, ABC, and HEOA, respectively. In addition, IHEOA improved the valid path rate by 11.1%, 3%, 7.5%, and 3% relative to the PSO, GWO, ABC, and HEOA, respectively. The planning paths and convergence curves in this model are shown in Figure 9.

The results indicate that the GA, PSO, GWO, and ABC algorithms exhibited varying degrees of local optimal entrapment during the optimization process, while the paths generated by the HEOA and its improved version, the IHEOA, were smoother with fewer inflection points. Although the GA algorithm demonstrated some robustness in the complex models, it was prone to local optima and slow convergence. The GWO and ABC performed well in simple models but lacked robustness in complex obstacle environments, struggling to consistently plan valid paths. While the PSO and the HEOA achieved relatively efficient and smooth path planning, the IHEOA outperformed them in terms of convergence accuracy and robustness, with faster convergence speed, particularly excelling in complex environments.

In conclusion, the Improved Human Evolutionary Optimization Algorithm (IHEOA) demonstrated excellent robustness and rapid convergence across the diverse environments, possessing strong global optimization capabilities. It efficiently planned smooth and effective paths, with a significant advantage in complex terrains.

## 6. Conclusions

To address the prevalent challenges of inadequate convergence accuracy and vulnerability to local optima in UAV three-dimensional path planning, this paper proposes an efficient path planning method based on an improved Human Evolutionary Optimization Algorithm. The proposed HEOA incorporates Logistic Chaotic Mapping integrated with an opposition-based learning strategy, introduces a guidance factor, and employs an adaptive *t*-distribution perturbation strategy, significantly improving the algorithm’s overall performance. Comparative experiments were conducted on various test functions using algorithms such as the IHEOA, CLB-HEOA, GF-HEOA, ATD-HEOA, and HEOA. The results demonstrate that the proposed improvement strategies resulted in substantial enhancements in precision, stability, and convergence speed for the three improved algorithms compared to the original HEOA, thereby validating the efficacy and advantages of these strategies. Notably, in both single-peak and multi-peak function tests, the IHEOA exhibited exceptional performance, indicating its effectiveness in efficient exploration and exploitation within complex environments.

Furthermore, the robustness of the improved IHEOA was validated through simulations in various environments under a multi-constraint path cost model. The experimental results showed that IHEOA possessed significant advantages in global optimization capabilities compared to the other algorithms, effectively avoiding local optima issues, and performed exceptionally well in complex environments with increased stability. The improved HEOA also displayed smoother trajectories and fewer inflection points in path planning, further underscoring its extensive application potential in UAV path planning.

In summary, the multi-constraint path cost model and the innovative IHEOA proposed in this paper offer an efficient and reliable solution for UAV path planning, showcasing substantial application prospects. Future research could focus on further optimizing the algorithm to continuously improve its performance, as well as exploring new application scenarios in multi-objective tasks, UAV swarm operations, and more complex environments for three-dimensional path planning. This endeavor will provide a more robust theoretical foundation and technical support for the practical application and advancement of UAV technology.

## Figures and Tables

**Table 1:** Test function specification.

Test Function	Range	**Dim**	**Min**
$F_{1}\left(x\right)=\sum_{i=1}^{n}x_{i}^{2}$	[−100, 100]	30	0
$F_{2}\left(x\right)=\max_{i}\left\{\left|x_{i}\right|,1\leq i\leq n\right\}$	[−100, 100]	30	0
$F_{3}\left(x\right)=\sum_{i=1}^{n}{\left(\left[x_{i}+0.5\right]\right)}^{2}$	[−100, 100]	30	0
$F_{4}\left(x\right)=\sum_{i=1}^{n}\left|x_{i}\right|+\prod_{i=1}^{n}\left|x_{i}\right|$	[−10, 10]	30	0
$F_{5}\left(x\right)=\sum_{i=1}^{n-1}\left[100{\left(x_{i+1}-{x_{i}}^{2}\right)}^{2}+{\left(x_{i}-1\right)}^{2}\right]$	[−30, 30]	30	0
$F_{6}\left(x\right)=\sum_{i=1}^{n}{\left(\sum_{j=1}^{i}x_{j}\right)}^{2}$	[−100, 100]	30	0
$F_{7}\left(x\right)=\frac{1}{4000}\sum_{i=1}^{n}x_{i}^{2}-\prod_{i=1}^{n}\cos\left(\frac{x_{i}}{\sqrt{i}}\right)+1$	[−600, 600]	30	0
$F_{8}\left(x\right)=-20\exp\left(-0.2\sqrt{\frac{1}{n}\sum_{i=1}^{n}x_{i}^{2}}\right)-\exp\left(\frac{1}{n}\sum_{i=1}^{n}\cos\left(2\pi x_{i}\right)\right)+20+e$	[−32, 32]	30	0
$F_{9}=\sum_{i=1}^{n}-x_{i}\sin\left(\sqrt{\left|x_{i}\right|}\right)$	[−500, 500]	30	−418.98 × Dim^n^
$F_{10}\left(x\right)=\sum_{i=1}^{n}\left[{x_{i}}^{2}-10\cos\left(2\pi x_{i}\right)+10\right]$	[−5.12, 5.12]	30	0
$\begin{array}{l} F_{11}\left(x\right)=\frac{\pi }{n}\left\{10\sin\left(\pi y_{1}\right)+\sum_{i=1}^{n-1}{\left(y_{i}-1\right)}^{2}\left[1+10\sin^{2}\left(\pi y_{i+1}\right)\right]+{\left(y_{n}-1\right)}^{2}\right\}\\ +\sum_{i=1}^{n}u\left(x_{i},10,100,4\right)\\ y_{i}=1+\frac{x_{i}+1}{4}\\ u\left(x_{i},a,k,m\right)=\left\{\begin{array}{l} k{\left(x_{i}-a\right)}^{m}\;\;x_{i}>a\\ 0\;\;\;\;\;-a<x_{i}<a\\ k{\left(-x_{i}-a\right)}^{m}\;\;x_{i}<-a \end{array}\right. \end{array}$	[−50, 50]	30	0
$\begin{array}{l} F_{12}\left(x\right)=0.1\left\{\sin^{2}\left(3\pi x_{1}\right)+\sum_{i=1}^{n}{\left(x_{i}-1\right)}^{2}\left[1+\sin^{2}\left(3\pi x_{i}+1\right)\right]\right.\\ \left.+{\left(x_{n}-1\right)}^{2}\left[1+\sin^{2}\left(2\pi x_{n}\right)\right]\right\}+\sum_{i=1}^{n}u\left(x_{i},5,100,4\right) \end{array}$	[−50, 50]	30	0

**Table 2:** Experimental results in test functions.

Function	Algorithm	Max	Min	Mean	Std
F1	HEOA	2.4671 × 10^−42^	6.4189 × 10^−60^	8.2541 × 10^−44^	2.0284 × 10^−85^
	CLB-HEOA	**1.1609** **× 10^−49^**	**4.5065** **× 10^−61^**	**4.07** **× 10^−51^**	**4.4814** **× 10^−100^**
	GF-HEOA	**9.9379** **× 10^−43^**	2.5677 × 10^−58^	4.5576 × 10^−44^	**3.4901** **× 10^−86^**
	ATD-HEOA	4.0677 × 10^−41^	1.0292 × 10^−59^	2.2295 × 10^−42^	6.3724 × 10^−83^
	IHEOA	**2.877** **× 10^−49^**	**3.3071** **× 10^−63^**	**1.1365** **× 10^−50^**	**2.7964** **× 10^−99^**
F2	HEOA	1.9976 × 10^−13^	6.7804 × 10^−17^	4.1112 × 10^−14^	3.414 × 10^−27^
	CLB-HEOA	**6.2768** **× 10^−15^**	**2.9536** **× 10^−21^**	**1.133** **× 10^−15^**	**4.6406 × 10** **^−30^**
	GF-HEOA	**1.3973 × 10** **^−13^**	**2.9685 × 10** **^−18^**	**2.2098 × 10** **^−14^**	**1.3222 × 10** **^−27^**
	ATD-HEOA	3.0482 × 10^−13^	**1.0939 × 10** **^−18^**	**3.6453e × 10** **^−14^**	4.5548 × 10^−27^
	IHEOA	**5.6437 × 10** **^−14^**	**1.2262 × 10** **^−21^**	**3.1744 × 10** **^−15^**	**1.2394 × 10** **^−28^**
F3	HEOA	3.8118	0.0030566	0.71778	0.92185
	CLB-HEOA	**0.051593**	**0.00091299**	**0.0096948**	**0.00016792**
	GF-HEOA	**3.0127**	0.00624	**0.62317**	**0.7349**
	ATD-HEOA	**2.2905**	0.0038808	**0.57117**	**0.47301**
	IHEOA	**0.018145**	**0.00083686**	**0.0052024**	**2.1021 × 10** **^−5^**
F4	HEOA	9.8708 × 10^−19^	6.1505 × 10^−30^	3.8224 × 10^−20^	3.2889 × 10^−38^
	CLB-HEOA	**2.2279 × 10** **^−26^**	**1.1079 × 10** **^−30^**	**2.1019 × 10** **^−27^**	**2.3734 × 10** **^−53^**
	GF-HEOA	8.1551 × 10^−18^	3.2226 × 10^−28^	2.7634 × 10^−19^	2.2146 × 10^−36^
	ATD-HEOA	1.1513 × 10^−17^	1.8408 × 10^−28^	4.0084 × 10^−19^	4.4091 × 10^−36^
	IHEOA	**1.8897 × 10** **^−26^**	**2.7243 × 10** **^−30^**	**1.3491 × 10** **^−27^**	**1.3596e × 10** **^−53^**
F5	HEOA	7.7192	0.66277	3.5703	3.1852
	CLB-HEOA	31.0691	0.90662	23.5837	101.8833
	GF-HEOA	17.585	**0.52163**	4.0238	17.5317
	ATD-HEOA	**7.6471**	**0.59903**	**2.7355**	**2.5589**
	IHEOA	28.7074	**0.2009**	22.4317	113.3637
F6	HEOA	4.7411 × 10^−58^	9.462 × 10^−64^	1.9048 × 10^−59^	7.4756 × 10^−117^
	CLB-HEOA	**8.6414 × 10** **^−59^**	**4.1181 × 10** **^−64^**	5.8003 × 10^−60^	**2.7256 × 10** **^−118^**
	GF-HEOA	**9.6796 × 10** **^−59^**	1.5808 × 10^−63^	5.2782 × 10^−60^	**3.3653 × 10** **^−118^**
	ATD-HEOA	**1.1483 × 10** **^−58^**	**3.792 × 10** **^−64^**	**1.1657 × 10** **^−59^**	**8.3052 × 10** **^−118^**
	IHEOA	7.6197 × 10^−58^	**1.9773 × 10** **^−64^**	2.8487 × 10^−59^	1.9244 × 10^−116^
F7	HEOA	0.76937	0	0.062458	0.021827
	CLB-HEOA	**0.24705**	0	**0.019838**	**0.0028784**
	GF-HEOA	**0.59618**	0	**0.069215**	**0.015512**
	ATD-HEOA	**0.20018**	0	**0.033655**	**0.0032499**
	IHEOA	**0.19416**	0	**0.044784**	**0.0029706**
F8	HEOA	8.7773 × 10^−10^	8.8818 × 10^−16^	4.2869 × 10^−11^	2.5869 × 10^−20^
	CLB-HEOA	**4.4409 × 10** **^−15^**	8.8818 × 10^−16^	**1.0066 × 10** **^−15^**	**4.2073 × 10** **^−31^**
	GF-HEOA	5.0744 × 10^−9^	8.8818 × 10^−16^	3.1493 × 10^−10^	1.1986 × 10^−18^
	ATD-HEOA	**2.9568 × 10** **^−10^**	8.8818 × 10^−16^	**2.4698 × 10** **^−11^**	**3.9662 × 10** **^−21^**
	IHEOA	**8.8818 × 10** **^−16^**	8.8818 × 10^−16^	**8.8818 × 10** **^−16^**	**0**
F9	HEOA	−5186.0789	−7214.4156	−6399.6368	284560.8141
	CLB-HEOA	**−5330.2659**	**−8485.4688**	−6377.8938	413384.7838
	GF-HEOA	**−5431.8474**	**−8376.8827**	**−6627.6507**	455791.5401
	ATD-HEOA	−5047.1905	**−7693.3983**	−6366.0274	563576.1488
	IHEOA	−4232.1402	**−8055.6087**	**−6451.858**	739154.4281
F10	HEOA	51.999	0	31.3531	62.6883
	CLB-HEOA	**34.3729**	0	**17.7222**	249.3872
	GF-HEOA	56.9311	30.0542	32.5621	**32.6846**
	ATD-HEOA	**45.8477**	0	**30.2301**	83.1312
	IHEOA	**32.1328**	0	**13.3742**	242.1269
F11	HEOA	1.753	3.2624 × 10^−5^	0.50867	0.20728
	CLB-HEOA	**0.0017046**	**1.6485 × 10^−5^**	**0.00025621**	**1.1817 × 10^−7^**
	GF-HEOA	3.5513	0.0068053	0.59293	0.71235
	ATD-HEOA	3.2479	0.0029736	**0.5052**	0.44449
	IHEOA	**0.85798**	2.1422 × 10^−5^	**0.02874**	**0.024529**
F12	HEOA	0.0079455	0.00052817	0.0026944	3.3177 × 10^−6^
	CLB-HEOA	0.08862	**0.00016096**	0.0053714	0.00026034
	GF-HEOA	0.010214	**0.00034436**	0.0035122	5.0556 × 10^−6^
	ATD-HEOA	0.011387	**0.0002815**	0.0028263	6.0678 × 10^−6^
	IHEOA	0.011539	**0.00015535**	**0.00233**	5.7731 × 10^−6^

**Table 3:** Performance indicators of various algorithms.

Environmental Model	Index	GA	PSO	GWO	ABC	HEOA	IHEOA
**10 peaks** Population Size: 50 Iterations: 60	Best	254.7174	241.4031	241.5485	249.8837	241.416	**241.1713**
	Mean	269.4059	244.8603	246.6655	279.5257	244.7827	**243.6999**
	Std	58.5727	**5.5188**	45.8681	406.6695	21.2761	26.7268
	15th-Best	/	/	/	/	241.6463	**241.21**
	Invnum-path	0	0	0	0	0	0
	Valid-rate	100%	100%	100%	100%	100%	100%
**20 peaks** Population Size: 50 Iterations: 100	Best	260.6816	248.6233	255.7319	258.6905	248.8133	**246.748**
	Mean	284.1838	253.3283	278.6418	326.9237	255.5482	**250.4231**
	Std	103.2585	**23.4995**	318.1155	2809.3019	243.6555	217.6289
	25th-Best	/	/	/	/	248.8168	**247.8806**
	Invnum-path	0	0	0	0	0	0
	Valid-rate	100%	100%	100%	100%	100%	100%
**30 peaks** Population Size: 50 Iterations: 100	Best	263.5978	244.0122	247.5378	254.9542	244.0805	**243.6575**
	Mean	284.0755	250.0702	272.4083	342.7798	247.2184	**244.5365**
	Std	172.4511	**155.9033**	1402.9837	8937.3567	2814.974	1105.5438
	25th-Best	/	/	/	/	246.5352	244.0971
	Invnum-path	**0**	3	1	2	1	**0**
	Valid-rate	**100%**	90%	97%	93%	97%	**100%**

## Data Availability

The data that support the findings of this study are available from the corresponding author upon request. There are no restrictions on data availability.

## References

[1] Yang L., Qi J., Xiao J., Yong X. (2014). A literature review of UAV 3D path planning. Proceedings of the 11th World Congress on Intelligent Control and Automation.

[2] Sun C.C., Jan G.E., Leu S.W., Yang K.C., Chen Y.C. (2015). Near-Shortest Path Planning on a Quadratic Surface with *O* (*n*\log *n*) Time. IEEE Sens. J..

[3] Yu Z., Si Z., Li X., Wang D., Song H. (2022). A novel hybrid particle swarm optimization algorithm for path planning of UAVs. IEEE Internet Things J..

[4] Penin B., Giordano P.R., Chaumette F. (2018). Minimum-time trajectory planning under intermittent measurements. IEEE Robot. Autom. Lett..

[5] Masehian E., Habibi G. (2007). Robot path planning in 3D space using binary integer programming. Int. J. Comput. Inf. Eng..

[6] Gong Q., Lewis L.R., Ross I.M. (2009). Pseudospectral motion planning for autonomous vehicles. J. Guid. Control. Dyn..

[7] Noto M., Sato H. (2000). A method for the shortest path search by extended Dijkstra algorithm. Proceedings of the SMC 2000 Conference Proceedings, 2000 IEEE International Conference on Systems, Man and Cybernetics. ‘Cybernetics Evolving to Systems, Humans, Organizations, and Their Complex Interactions’, (Cat. No. 0).

[8] Cai Y., Xi Q., Xing X., Gui H., Liu Q. (2019). Path planning for UAV tracking target based on improved A-star algorithm. Proceedings of the 2019 1st International Conference on Industrial Artificial Intelligence (IAI).

[9] Ren X., Tan L., Jiaqi S., Lian X. (2021). Multi-target UAV path planning based on improved RRT algorithm. J. Phys. Conf. Ser..

[10] Karaman S., Walter M.R., Perez A., Frazzoli E., Teller S. (2011). Anytime motion planning using the RRT. Proceedings of the 2011 IEEE International Conference on Robotics and Automation.

[11] Pan Z., Zhang C., Xia Y., Xiong H., Shao X. (2021). An improved artificial potential field method for path planning and formation control of the multi-UAV systems. IEEE Trans. Circuits Syst. II Express Briefs.

[12] Gasparetto A., Boscariol P., Lanzutti A., Vidoni R. (2015). Path planning and trajectory planning algorithms: A general overview. Motion and Operation Planning of Robotic Systems: Background and Practical Approaches.

[13] Kesavan V., Kamalakannan R., Sudhakarapandian R., Sivakumar P. (2020). Heuristic and meta-heuristic algorithms for solving medium and large scale sized cellular manufacturing system NP-hard problems: A comprehensive review. Mater. Today Proc..

[14] Tang J., Liu G., Pan Q. (2021). A review on representative swarm intelligence algorithms for solving optimization problems: Applications and trends. IEEE/CAA J. Autom. Sin..

[15] Deng L., Chen H., Zhang X., Liu H. (2023). Three-dimensional path planning of UAV based on improved particle swarm optimization. Mathematics.

[16] Zhang R., Li S., Ding Y., Qin X., Xia Q. (2022). UAV path planning algorithm based on improved Harris Hawks optimization. Sensors.

[17] Kumar R., Singh L., Tiwari R. (2023). Novel reinforcement learning guided enhanced variable weight grey wolf optimization (RLV-GWO) algorithm for multi-UAV path planning. Wirel. Pers. Commun..

[18] Dewangan R.K., Saxena P. (2023). Three-dimensional route planning for multiple unmanned aerial vehicles using Salp Swarm Algorithm. J. Exp. Theor. Artif. Intell..

[19] Wang W., Ye C., Tian J. (2023). SGGTSO: A Spherical Vector-Based Optimization Algorithm for 3D UAV Path Planning. Drones.

[20] Chen H., Liang Y., Meng X. (2023). A UAV Path Planning Method for Building Surface Information Acquisition Utilizing Opposition-Based Learning Artificial Bee Colony Algorithm. Remote Sens..

[21] Wu X.J., Xu L., Zhen R., Wu X.L. (2023). Global and local moth-flame optimization algorithm for UAV formation path planning under multi-constraints. Int. J. Control. Autom. Syst..

[22] Qadir Z., Zafar M.H., Moosavi S.K.R., Le K.N., Mahmud M.P. (2021). Autonomous UAV path-planning optimization using metaheuristic approach for predisaster assessment. IEEE Internet Things J..

[23] Qadir Z., Zafar M.H., Moosavi S.K.R., Le K.N., Tam V.W. (2022). Optimizing UAV path for disaster management in smart cities using metaheuristic algorithms. Computational Intelligence for Unmanned Aerial Vehicles Communication Networks.

[24] Lian J., Hui G. (2024). Human evolutionary optimization algorithm. Expert Syst. Appl..

[25] Zhang D., Wang Z., Sun F. (2024). Somersault Foraging and Elite Opposition-Based Learning Dung Beetle Optimization Algorithm. Appl. Sci..

[26] Ma W., Yu T., Wang Z., Li X. (2023). A novel image encryption scheme based on Logistic cosine cascade maps. Phys. Scr..

[27] Yin S., Luo Q., Du Y., Zhou Y. (2022). DTSMA: Dominant swarm with adaptive *t*-distribution mutation-based slime mould algorithm. Math. Biosci. Eng..

[28] Kanso A., Smaoui N. (2009). Logistic chaotic maps for binary numbers generations. Chaos Solitons Fractals.

[29] Xu B., Ye X., Wang G., Huang Z., Zhang C. (2023). A Fractional-Order Improved Quantum Logistic Map: Chaos, 0–1 Testing, Complexity, and Control. Axioms.

[30] Yao X., Liu Y., Lin G. (1999). Evolutionary programming made faster. IEEE Trans. Evol. Comput..

[31] Liang J.J., Qu B.Y., Suganthan P.N. (2013). Problem Definitions and Evaluation Criteria for the CEC 2014 Special Session and Competition on Single Objective Real-Parameter Numerical Optimization.

